# IFN-α/β/IFN-γ/IL-15 pathways identify GBP1-expressing tumors with an immune-responsive phenotype

**DOI:** 10.1007/s10238-024-01328-w

**Published:** 2024-05-17

**Authors:** Lei Wang, Yuxuan Wei, Zheng Jin, Fangfang Liu, Xuchang Li, Xiao Zhang, Xiumei Bai, Qingzhu Jia, Bo Zhu, Qian Chu

**Affiliations:** 1 Department of Oncology, Tongji Hospital, Huazhong University of Science and Technology, Wuhan, 430030 Hubei People’s Republic of China; 2 Institute of Life Sciences, Chongqing Medical University, Chongqing, 400032 People’s Republic of China; 3 Research Institute, GloriousMed Clinical Laboratory (Shanghai) Co., Ltd, Shanghai, 201318 People’s Republic of China; 4 Army 953 Hospital, Shigatse Branch of Xinqiao Hospital, Army Medical University, Shigatse, 857000 People’s Republic of China; 5 Department of Oncology, Xinqiao Hospital, Army Medical University, Chongqing, 400037 People’s Republic of China; 6 Chongqing Key Laboratory of Immunotherapy, Chongqing, 400037 People’s Republic of China

**Keywords:** GBP1, Tumor microenvironment, Immunotherapy, Biomarker

## Abstract

Immunotherapy is widely used in cancer treatment; however, only a subset of patients responds well to it. Significant efforts have been made to identify patients who will benefit from immunotherapy. Successful anti-tumor immunity depends on an intact cancer-immunity cycle, especially long-lasting CD8^+^ T-cell responses. Interferon (IFN)-α/β/IFN-γ/interleukin (IL)-15 pathways have been reported to be involved in the development of CD8^+^ T cells. And these pathways may predict responses to immunotherapy. Herein, we aimed to analyze multiple public databases to investigate whether IFN-α/β/IFN-γ/IL-15 pathways could be used to predict the response to immunotherapy. Results showed that IFN-α/β/IFN-γ/IL-15 pathways could efficiently predict immunotherapy response, and guanylate-binding protein 1 (*GBP1*) could represent the IFN-α/β/IFN-γ/IL-15 pathways. In public and private cohorts, we further demonstrated that *GBP1* could efficiently predict the response to immunotherapy. Functionally, *GBP1* was mainly expressed in macrophages and strongly correlated with chemokines involved in T-cell migration. Therefore, our study comprehensively investigated the potential role of GBP1 in immunotherapy, which could serve as a novel biomarker for immunotherapy and a target for drug development.

**Supplementary Information:**

The online version contains supplementary material available at 10.1007/s10238-024-01328-w.

## Introduction

Recently, immunotherapy targeting the PD-1/PD-L1 pathway has significantly improved T-cell-mediated anti-tumor responses and induced objective clinical responses in multiple tumor types. However, only a minority of patients experience durable survival after treatment with therapies. To maximize treatment benefits, predictive biomarkers, such as PD-L1 and tumor mutational burden (TMB), have been developed to guide therapeutic decisions [1]. However, data from clinical trials have demonstrated that patients with low PD-L1 expression could still respond to immunotherapy [2, 3]. In addition, currently used single biomarkers ignore the complex immune responses that occur in the tumor microenvironment (TME). Hence, precise prediction of immunotherapy requires further investigation.

The TME plays a crucial role in cancer progression and therapeutic responses [4]. Tumor-infiltrating immune cells, especially CD8^+^ T cells, can profoundly influence tumor progression and success of anticancer therapies [5]. Previously, T-cell infiltration has been linked to the response to immunotherapy, specifically the immune checkpoint blockade (ICB) [6, 7]. Successful and long-term immunotherapeutic responses depend on CD8^+^ T-cell activation, cancer cell killing, and memory CD8^+^ T-cell maintenance [8]. These steps require distinct signaling pathways: (1) Interferon (IFN)-α/β pathway: Type I IFN could boost cDC1 cross-priming and CD8^+^ T-cell reactivation [9]; (2) IFN-γ pathway: Cytotoxic CD8^+^ T cells typically secret large amounts of IFN-γ to kill tumorigenic cells [10]; and (3) interleukin (IL)-15 pathway: IL-15 signaling provides a sustained immune response and maintains the memory phenotype of CD8^+^ T cells [11, 12]. Disruption of these steps may hinder the enduring effects of immunotherapy. Therefore, a comprehensive understanding of the TME could help improve immunotherapeutic strategies.

To address this need, we analyzed the IFN-α/β/IFN-γ/IL-15 pathways to identify a potential combinatorial biomarker to predict responses to ICB. Bulk and single-cell RNA-seq data were collected and analyzed from multiple studies and cancer types, and results showed that a single IFN-α/β/IFN-γ/IL-15 pathway could predict clinical responses and survival to ICB. We found that *GBP1* could represent and positively correlate with these three pathways, where its expression also positively correlated with the hot immune microenvironment. Further analysis showed that GBP1 mRNA and protein expression in baseline tumor tissues correlated with improved response and overall survival (OS) in multiple cohorts. Cumulatively, this study revealed that GBP1 could serve as a predictive biomarker and may improve the predictive outcomes of patients receiving ICB.

## Materials and methods

### Data collection of bulk and single-cell RNA-seq cohorts

Bulk RNA-seq data from four public immune checkpoint inhibitor (ICI)-treated melanoma cohorts [13–16] that contained 286 pretreated patients were collected to validate the association between selected signaling pathways, *GBP1*, and the efficacy of immunotherapy. All patients with melanoma were treated with anti-PD-1 or anti-CTLA-4 antibodies. To calculate the correlation between selected signaling pathways and *GBP1* expression in non-small cell lung cancer (NSCLC), four public cohorts with 417 patients with NSCLC were retrieved from the Gene Expression Omnibus (GEO) database (accession numbers: GSE81089, GSE103584, GSE112996, and GSE181820). In addition, transcriptomic data from 10,148 cases comprising 33 tumor types were downloaded from The Cancer Genome Atlas (TCGA) to investigate the correlation between selected signaling pathways and *GBP1* expression, as well as the TME.

To evaluate the abundance of *GBP1* in various cell populations, two cohorts of NSCLC [17] and melanoma [18], and a pan-cancer cohort of 13 tumor types [19], were used in this study.

### Definition of the enrichment of selected signaling pathways

Three anti-tumor immune signaling pathways, including INF-α/β (REACTOME_INTERFERON_ALPHA_BETA_SIGNALING), INF-γ (BIOCARTA_IFNG_PATHWAY), and IL-15 (REACTOME_INTERLEUKIN_15_SIGNALING), were derived from the MSigDB database [20, 21] and quantified via single-sample Gene Set Enrichment Analysis (ssGSEA) [22]. In each cohort, patients were classified into high and low groups using the median enrichment score of each pathway as the threshold. The pooled cohort comprised the group information from each cohort.

### Identification of GBP1 as an indicator for selected signaling pathways

To explore whether the activity within three selected signaling pathways could be represented by a single gene. The ssGSEA scores of above three selected signaling pathways were calculated for each tumor sample. Subsequently, Pearson’s correlation coefficient was employed to assess the relationship between the expression level of each gene and the ssGSEA score corresponding to each chosen pathway across various tumor types. For each gene, the number of tumor types demonstrating a significantly positive correlation coefficient (*r* > 0 and *p* value ≤ 0.05) was aggregated. The genes were ranked based on the quantity of tumor types they were significantly associated with.

### Survival and treatment response analysis

For the ICI-treated cohorts, patients were classified into high and low groups using the median enrichment scores of three selected signaling pathways, the median expression value of *GBP1*, or the median ssGSEA scores of six hot-/cold-related gene sets [23–28]. The pooled cohort comprised the group information from each cohort. Survival curves were generated using the Kaplan–Meier method with survival package v.2.44 and visualized using the survminer package. Statistical significance between two groups was assessed using the log-rank test. Patients who survived longer than 36 months were defined as having long-term survival, and the percentage of patients with long-term survival analyzed.

The ICI treatment response was defined based on RECIST1.1. The objective response rate (ORR) was defined as the percentage of patients who achieved a complete or partial response. Responders were defined as patients who achieved an objective response or maintained stable disease for more than 6 months. The ORR and percentage of responders were analyzed in the individual ICI-treated and pooled cohorts.

### Correlation analysis

For the TCGA data, GEO cohorts, and ICI-treated cohorts, the ssGSEA scores of the three selected signaling pathways were calculated for each tumor sample. The expression levels of *GBP1*, *CXCL9*, *CXCL10*, and *CCL5* were normalized using log transformation. Correlation coefficients were calculated using Pearson’s correlation coefficient.

### Tumor microenvironment analysis

For TCGA data, patients were initially classified according to the median expression level of *GBP1* in each tumor type. Thereafter, classification information for each tumor type was summarized to assign patients to high and low groups. The high group represented patients with a higher transcriptomic level of *GBP1* than that of the median for each tumor type, and the low group represented patients with a lower transcriptomic level of *GBP1* than that of the median.

For the “pro-to-anti-inflammatory cytokine ratio,” the ratio of the average expression level (log-transformed FPKM value) of pro-inflammatory cytokines (IFN-γ, IL-1, and IL-2) versus anti-inflammatory cytokines (TGFB1, IL-10, IL-4, and IL-11) was calculated in each tumor sample. To calculate the ratio of CD8^+^ T cells versus Tregs, M1 macrophages, and myeloid-derived suppressor cells (MDSCs), the ratio of the average expression levels (log-transformed FPKM value) of the gene set for the corresponding cell types was calculated.

To determine the “hot” or “cold” microenvironment for the high and low groups, six hot-/cold-related gene sets were collected from the public databases used in this study. Scores were calculated using ssGSEA.

The Wilcoxon signed-rank test was used to compare the distribution of values between tumors with high and low *GBP1* expression levels. The calculated ratios or scores of each tumor sample were standardized among samples from the same tumor type.

### Single-cell RNA-seq analysis

The human melanoma and NSCLC scRNA-seq datasets were downloaded from the GEO database (accession numbers: GSE115978 and GSE117570). Quality control measures were applied to the cells. After filtering, the UMI count matrix was normalized by using the “NormalizeData” function with default parameters. For the clustering of all cell types, 50 principal component analyses were applied to reduce dimensionality using the “FindClusters” function on 50 principal components (PCs) with a 1.2 resolution in GSE115978 and 0.4 in GSE117570, and each cluster annotated with known markers. Uniform Manifold Approximation and Projection (UMAP) using the “RunUMAP” function was performed for visualization in two dimensions with the same PCs. *GBP1* expression was visualized via feature and violin plots using the “FeaturePlot” and “VlnPlot” functions in all cell types.

For the pan-cancer cohort, raw count data were normalized using the “NormalizedData” function from Seurat [29] with default parameters. Cell-type information for each cell was obtained from the public databases. Violin plots of the normalized expression levels of *GBP1* in each cell type were obtained using the “VlnPlot” function.

### Enrichment analysis

For TCGA-LUAD, TCGA-LUSC, and TCGA-SKCM, patients were classified into two groups based on the median FPKM value of *GBP1*. Differentially expressed genes (DEGs) were calculated between the groups for each tumor type using DESeq2 [30] with raw count data. Significant genes were defined when |log2FoldChange|≥ 2 and adjusted *p* value ≤ 0.05. Gene ontology enrichment analysis of the biological processes of DEGs was performed using the clusterProfiler package [31].

### Clinical features of tissue microarrays

Two cohorts of patients with NSCLC were included in this study. Cohort 1 contained 253 paraffin-embedded tumor tissues retrospectively collected at the Department of Oncology and Department of Pathology, Tongji Hospital (Wuhan, China). Cohort 2 retrospectively acquired baseline tumor tissues from 128 patients treated with immunotherapies-based treatments and followed-up with CT imaging to evaluate treatment response and survival. The treatment response was evaluated based on iRECIST [32]. The study was approved by the ethics board of Tongji Hospital, Huazhong University of Science and Technology (TJIRB20200731). Informed consent for this retrospective analysis was waived.

### Immunohistochemistry assays

The sections were deparaffinized with xylene and rehydrated in 100, 90, 80, and 70% ethanol for 10 min. The slides were then rinsed under running distilled water for 3 min. Antigen retrieval was processed with steaming slides in sodium citrate buffer heated at 99–100 °C for 20 min. The slides were removed from the heat and allowed to stand at room temperature in the buffer for 20 min. Endogenous peroxidase activity was analyzed using 3% hydrogen peroxide. After washing with 1 × TBST, a universal protein block was applied with bovine serum albumin. Then, the slides were stained with the monoclonal mouse anti‑Human GBP1 Antibody (1:200, clone 4D10, LS‑B4320; LSBio, Inc., Seattle, WA, USA) and the recombinant rabbit monoclonal to CD8 alpha antibody (1:1000, clone EPR22483-288, ab245118; Abcam, Cambridge, MA, USA) in phosphate-buffered saline and incubated at 4 °C overnight. The next day, the sections were cooled naturally to room temperature and incubated with the HRP-conjugated secondary antibody against the primary antibody. Then, the slides were immunostained with a peroxidase/diaminobenzidine (DAB) rabbit/mouse Bio Detection Kit (Kit-9710, DAB-0031; Maixin-Bio, Fuzhou, China). Subsequently, the sections were counterstained with hematoxylin (Zymed Laboratories, San Francisco, CA, USA) for 5 min and covered with a coverslip. The slides were scanned using CaseViewer software. DAB stain intensities were analyzed using the CaseViewer TMA function. The relationship between GBP1 and CD8^+^ T-cell infiltration was assessed using the Pearson method. Overall survival was analyzed using Kaplan–Meier curves with the log-rank test.

### Statistical analysis

Differences between two groups were analyzed using Student’s *t*-test. Univariate Cox analysis was used to define the hazard ratio of *GBP1* in the ICI-treated cohorts. Statistical significance was set at a *p* value < 0.05. Analyses were conducted using R (version 4.0.5) and GraphPad (version 8.0) software.

## Results

### IFN-α/β/IFN-γ/IL-15 pathways correlate with improved clinical response and overall survival in patients receiving immune checkpoint blockade

**Fig. 1:**
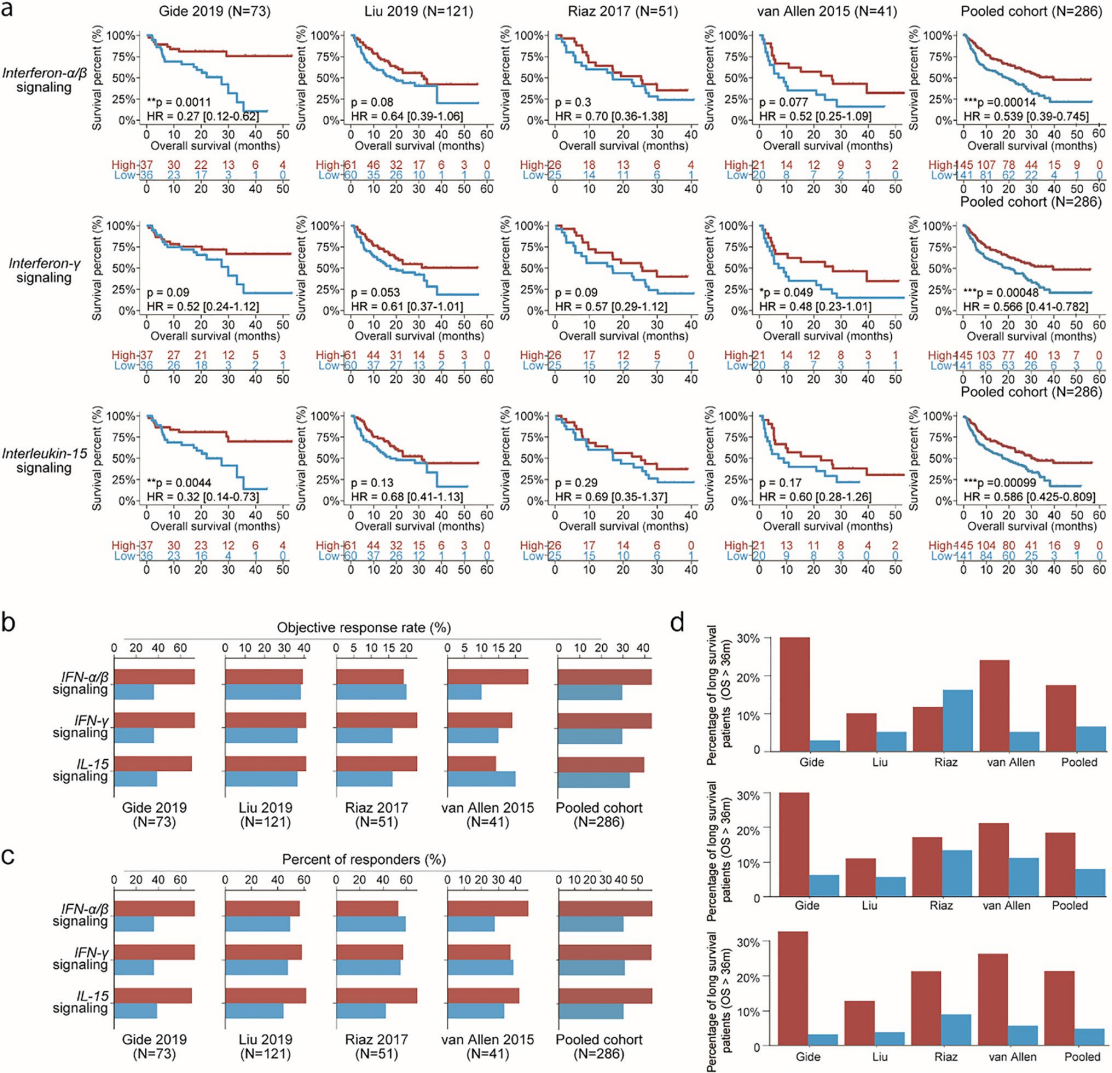
Expression of IFN-α/β/IFN-γ/IL-15 pathways is associated with prolonged survival rates and improved clinical response. **a** Kaplan–Meier estimates of overall survival (OS) were stratified according to expression of the IFN-α/β/IFN-γ/IL-15 pathways in the four immunotherapy and pooled cohorts. **b** Objective response rates (ORRs) were observed in the four immunotherapy and pooled cohorts for each group. **c** Percentages of responders segregated by expression of the IFN-α/β/IFN-γ/IL-15 pathways. **d** Percentage of patients with long-term survival was calculated for the four immunotherapy cohorts based on *GBP1* expression

To determine whether the IFN-α/β/IFN-γ/IL-15 pathways can be used as a tool to predict survival and response to ICB, we analyzed four cohorts with patients who had received ICB therapy, including the Gide (melanoma, *n* = 73) [13], Liu (melanoma, *n* = 121) [14], Riaz (melanoma, *n* = 51) [15], and van Allen (melanoma, *n* = 41) [16] cohorts (Fig. 1a). In the Gide cohort, the high-expression IFN-α/β pathway group had a significantly lower hazard ratio (HR = 0.27 [0.12–0.62], *p* = 0.0011) compared with that in the low-expression group. In other cohorts, we also observed a numerically decreased hazard ratio following ICB immunotherapy. The IFN-γ pathway displayed a significant positive association with a longer OS in the van Allen cohort (HR = 0.48 [0.23–1.01], *p* = 0.049), whereas the IL-15 pathway predicted a longer OS in the Gide cohort (HR = 0.32 [0.14–0.73], *p* = 0.0044). We further investigated the predictive effects of these three pathways through analysis of the pooled cohorts (*n* = 286). Collectively, all three pathways indicated a significantly lower hazard ratio in the pooled cohorts (IFN-α/β pathway: HR = 0.539 [0.39–0.745], *p* = 0.00014; IFN-γ pathway: HR = 0.566 [0.41–0.782], *p* = 0.00048; and IL-15 pathway: HR = 0.586 [0.425–0.809], *p* = 0.00099). Prolonged survival was also observed when all three pathways were highly expressed in these cohorts (Supplement Figure S1).

Next, we investigated the impact that the IFN-α/β/IFN-γ/IL-15 pathways have on tumor shrinkage. Here, we introduced the objective response to evaluate the magnitude of tumor shrinkage. Patients in each cohort were classified into responders and non-responders based on outcome measurements (‘‘responder’’ is defined as a complete response [CR] or PR, and ‘‘non-responder’’ defined as SD or PD [33]). Almost all high-expression pathway groups had a higher ORR than that in the low-expression group (Fig. 1b). Similar to the ORR, the percentage of responders was higher in the high-expression group (Fig. 1c). These results indicate that higher expression of these pathways may support a better clinical response. Sustained effects of immunotherapy are crucial for long-term survival. We then investigated whether the IFN-α/β/IFN-γ/IL-15 pathways could predict long-term survival. Patients with higher expression of the three pathways displayed a higher OS of > 36 months (Fig. 1d). Cumulatively, these data suggest that the IFN-α/β/IFN-γ/IL-15 pathways could predict the response and survival in patients treated with ICB.

### *GBP1* positively correlates with the IFN-α/β/IFN-γ/IL-15 pathways

**Fig. 2:**
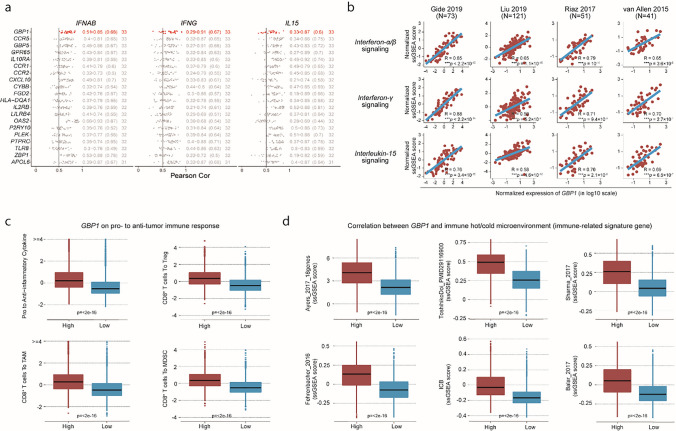
*GBP1* represents the IFN-α/β/IFN-γ/IL-15 pathways. **a** The correlation of indicated genes with the IFN-α/β/IFN-γ/IL-15 pathways was ranked by using Pearson’s correlation coefficient based on The Cancer Genome Atlas (TCGA) dataset. **b** Pearson’s correlation coefficient of *GBP1* expression with that of the IFN-α/β/IFN-γ/IL-15 pathways in the immunotherapy cohorts. **c** Ratios of the pro- to anti-inflammatory cytokines and CD8^+^ T cells to Tregs, tumor-associated macrophages (TAMs), and myeloid-derived suppressor cells (MDSCs) in the high and low *GBP1* expression groups. **d** Immune checkpoint blockade (ICB) responsiveness score in the high and low *GBP1* expression groups

As each pathway contains multiple genes, it is inconvenient and costly to fully examine them in clinical practice. Thus, we aimed to identify a single gene that could represent these three pathways. We analyzed transcriptomes from the TCGA database. The correlation coefficients between each gene and the three pathways were calculated for 33 cancer types. In the list, *GBP1* was found to rank first (Fig. 2a). GBP1 is a GTPase belonging to the dynamin superfamily, which could be provoked by IFN-γ signaling [34]. As CD8^+^ T-cell is the primary producer of IFN-γ, the expression of GBP1 in the context of immunotherapy may reflect the active adaptive immunity. This finding supports the positive correlation results obtained in this study. Then, we confirmed the association between *GBP1* and the IFN-α/β/IFN-γ/IL-15 pathways in the immunotherapy cohorts. *GBP1* was positively correlated with IFN-α/β/IFN-γ/IL-15 pathways (allρ > 0.5, *p* < 0.001) (Fig. 2b). To extend this finding, we confirmed this in the GEO datasets, which also showed that *GBP1* was positively correlated with the IFN-α/β/IFN-γ/IL-15 pathways (Supplement Figure S2). This suggests that the positive correlation observed is likely a universal phenomenon.

IFN-α/β/IFN-γ/IL-15 pathways are involved in the development of CD8^+^ T cells [11], and we have found that *GBP1* positively correlated with them. Thus, we aimed to characterize the immune context of the TME with *GBP1* expression. It has been reported that the ratio of pro-inflammatory cytokines to immunosuppressive cytokines and the ratio of CD8^+^ T cells to Tregs, tumor-associated macrophages (TAMs), and MDSCs are related to the responsiveness to ICB immunotherapy [35]. Pro-inflammatory and immunosuppressive cytokines were significantly elevated in tumors with high *GBP1* expression, which suggests that GBP1 could support anti-tumor immunity. Further investigation revealed that the ratio of CD8^+^ T cells to Tregs, TAMs, and MDSCs was significantly higher in tumors with high *GBP1* expression (Fig. 2c). This suggests that GBP1 could promote the infiltration of CD8^+^ T cells, thereby creating an immuno-supporting microenvironment. GSEA supported these findings, showing that *GBP1* enriched IL-2 signaling and the inflammatory response, indicating an activated immune response (Supplement Figure S3). We further applied multiple gene signatures to evaluate the responsiveness to immunotherapy, which revealed that patients with high *GBP1* expression tended to be more sensitive to immunotherapy (Fig. 2d). Collectively, the results suggest that *GBP1* expression could be used to represent the IFN-α/β/IFN-γ/IL-15 pathways, and that *GBP1* indicates an immuno‑hot TME.

### *GBP1* expression in baseline tumor tissues correlates with improved overall survival and treatment response

**Fig. 3:**
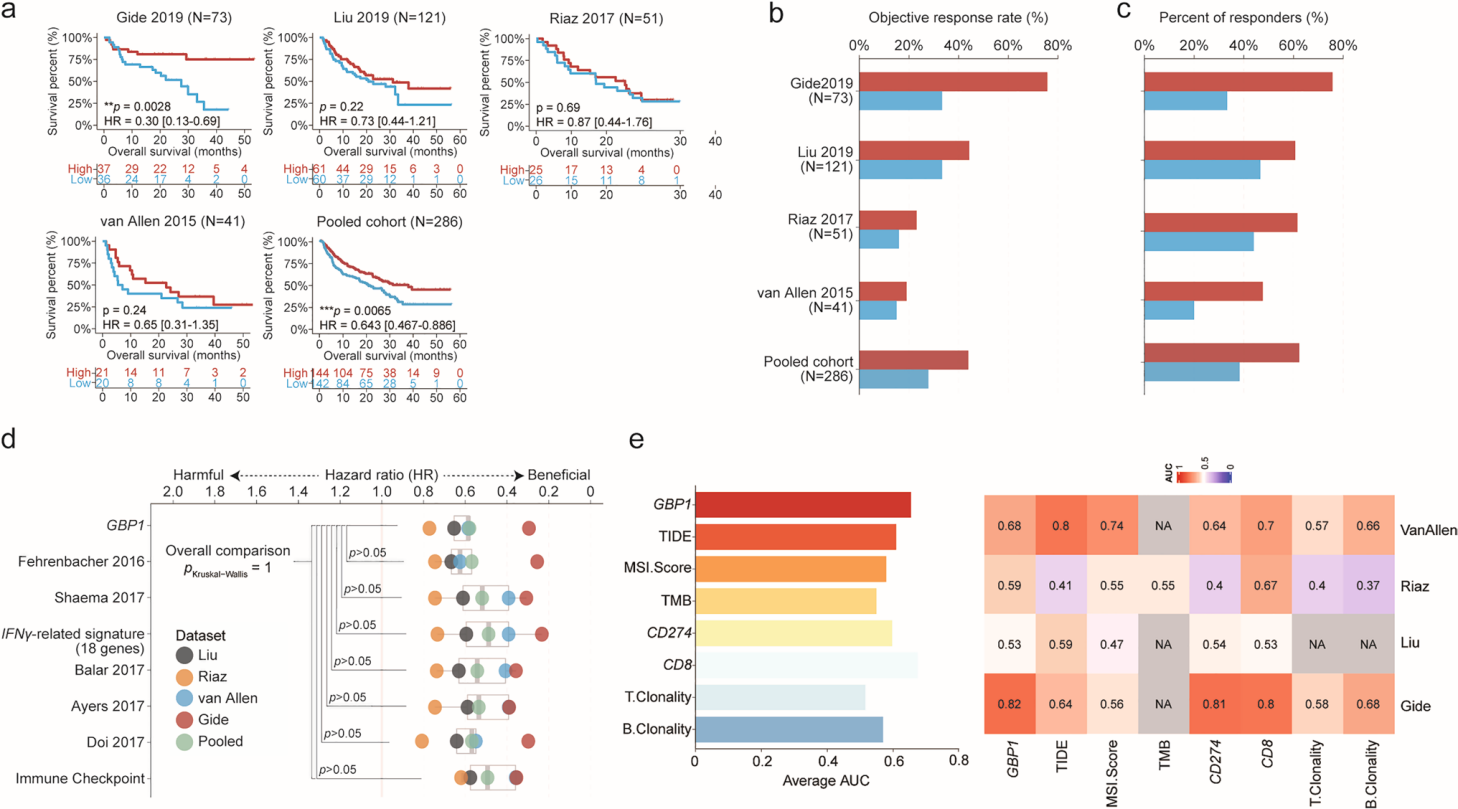
*GBP1* in the baseline tumor tissue correlates with favorable responses to immune checkpoint blockade. **a** Overall survival (OS) of patients treated with immune checkpoint inhibitors according to *GBP1* expression. **b** Objective response rates (ORRs) were observed in the immunotherapy and pooled cohorts for each group. **c** Percentage of responders segregated by *GBP1* expression. **d** Comparison of the hazard ratio of *GBP1* and reported predictive markers. **e** Comparison of AUC values of *GBP1* and representative markers (TIDE, MSI score, TMB, CD274, CD8, and B-/T-cell clonality)

IFN-α/β/IFN-γ/IL-15 pathways were associated with improved survival and response in patients treated with ICIs, and *GBP1* could serve as a surrogate to IFN-α/β/IFN-γ/IL-15 activity in pan-cancer scale analysis. We investigated whether *GBP1* alone produced similar results. We analyzed survival differences between *GBP1*^hi^ and *GBP1*^lo^ patients in the immunotherapy cohorts (Fig. 3a). Patients expressing higher *GBP1* mRNA levels exhibited significantly prolonged survival rates in the Gide cohort (HR = 0.30 [0.13–0.69], *p* = 0.0028). In the Liu, Riaz, and van Allen cohorts, higher *GBP1* levels tended to be associated with longer survival, but did not reach statistical significance. In the pooled cohort, *GBP1* showed significantly prolonged survival rates. The clinical response was further compared between the high and low *GBP1* groups. In all cohorts, the ORR was much higher in the high *GBP1* group (Fig. 3b), which also had more responders than those in the low *GBP1* group (Fig. 3c). Together, these results show that *GBP1* could predict survival and clinical response in patients treated with ICB, and the predictive effect was comparable with that of the IFN-α/β/IFN-γ/IL-15 pathways.

Multiple studies have reported that immune scores and immune-related signatures are predictive biomarkers for clinical responses [36–38]. To determine the universal predictive and prognostic potential of *GBP1* in comparison with other methods, we analyzed the hazard ratio of *GBP1* and existing scoring systems and signatures in the immunotherapy cohorts [23, 24, 26]. The hazard ratio of *GBP1* was comparable with those of these signatures, indicating that *GBP1* had a comparable predictive effect (Fig. 3d). We further analyzed the area under the receiver operating characteristic curve (AUC) values for *GBP1* expression and existing biomarkers, including the most commonly used biomarker, CD274 and TMB. The average AUC of *GBP1* was higher than that of CD274 and TMB (Fig. 3e). The comparative analysis of GBP1 with the existing biomarkers suggested that GBP1 could serve as a potential indicator of immunotherapy. Furthermore, GBP1 is a single biomarker that is easily detected, thus making it more applicable in clinical practice than existing immune-related signatures.

### *GBP1* is mainly expressed in macrophages and associated with functional T cells

**Fig. 4:**
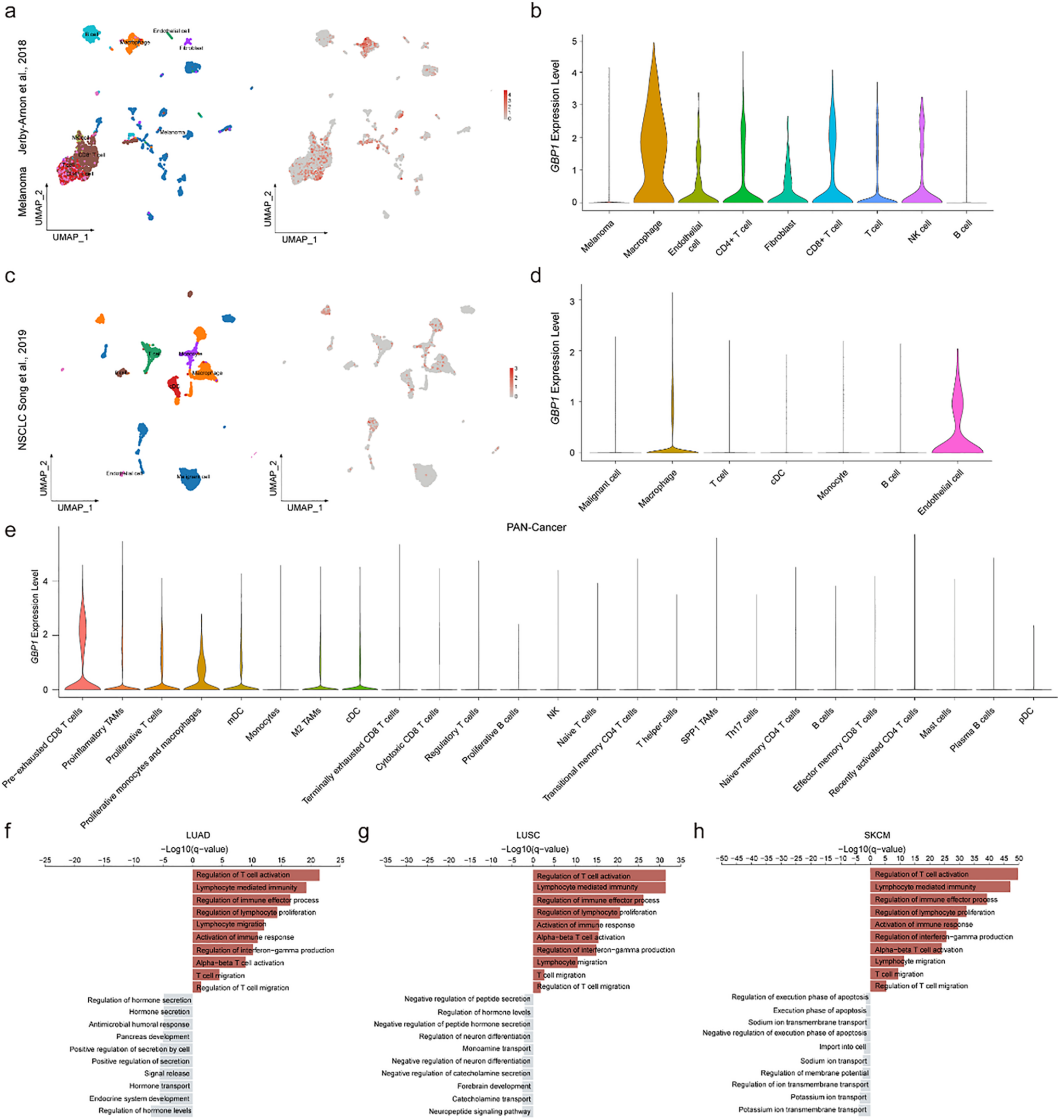
*GBP1* is mainly expressed in macrophages and correlated with T-cell migration and activation. **a** Uniform Manifold Approximation and Projection (UMAP) and **b** violin plots displaying cell-type annotations and *GBP1* expression in single cells of the melanoma patient cohort. **c** UMAP and **d** violin plots exhibiting cell-type annotations and *GBP1* expression in single cells of the NSCLC cohort. **e** Violin plot of *GBP1* expression in immune subtypes from the pan-cancer analysis. **f**–**h** Gene Set Enrichment Analysis (GSEA) results showing the most significantly enriched pathways in the LUAD (**f**), LUSC (**g**), and SKCM (**h**) patient groups

GBP1 is involved in the host immune response to exotic cells and can be induced by IFN expression [34]. The role of GBP1 in tumors is complex, and both anti- and pro-tumor effects have been reported. To determine the specific cell type expressing GBP1 that contributes to immunotherapy-responsive tumors, we collected published single-cell RNA sequencing data from patients with melanoma and NSCLC. In a melanoma dataset [18], *GBP1* was rarely expressed in tumor cells, whereas it was comparably enriched in macrophages, as well as CD8^+^ T, CD4^+^ T, NK, and endothelial cells (Fig. 4a and b). In the NSCLC datasets [17], we found a similar expression pattern, with macrophages and endothelial cells displaying higher *GBP1* expression levels (Fig. 4c and d). These results suggest that *GBP1* is seldom expressed in tumor cells, but highly expressed in immune cells. To identify cell types expressing *GBP1*, we conducted a pan-cancer analysis, including melanoma and NSCLC, to evaluate *GBP1* expression in 25 immune cell types. Similarly, *GBP1* was mainly expressed in monocytes and macrophages (Fig. 4e and Supplement Figure S4). Macrophages have been reported to account for a large proportion of the immune cells present in the TME. These results suggest that macrophages are the primary cell type that expresses *GBP1*. To elucidate the function of tumors highly expressing *GBP1*, we conducted functional analysis. GSEA showed that T-cell migration and activation were significantly upregulated across multiple tumors (Fig. 4f–h). T-cell infiltration of the TME depends on key chemokines. And macrophage-derived CXCL9 and CXCL10 are important for ICB-induced anti-tumor immunity [39]. The results of GSEA, together with the previous findings, suggested that GBP1 expression in tumors may promote CD8^+^ T-cell migration.

### *GBP1* is positively correlated with key chemokines that direct CD8^+^ T-cell migration

**Fig. 5:**
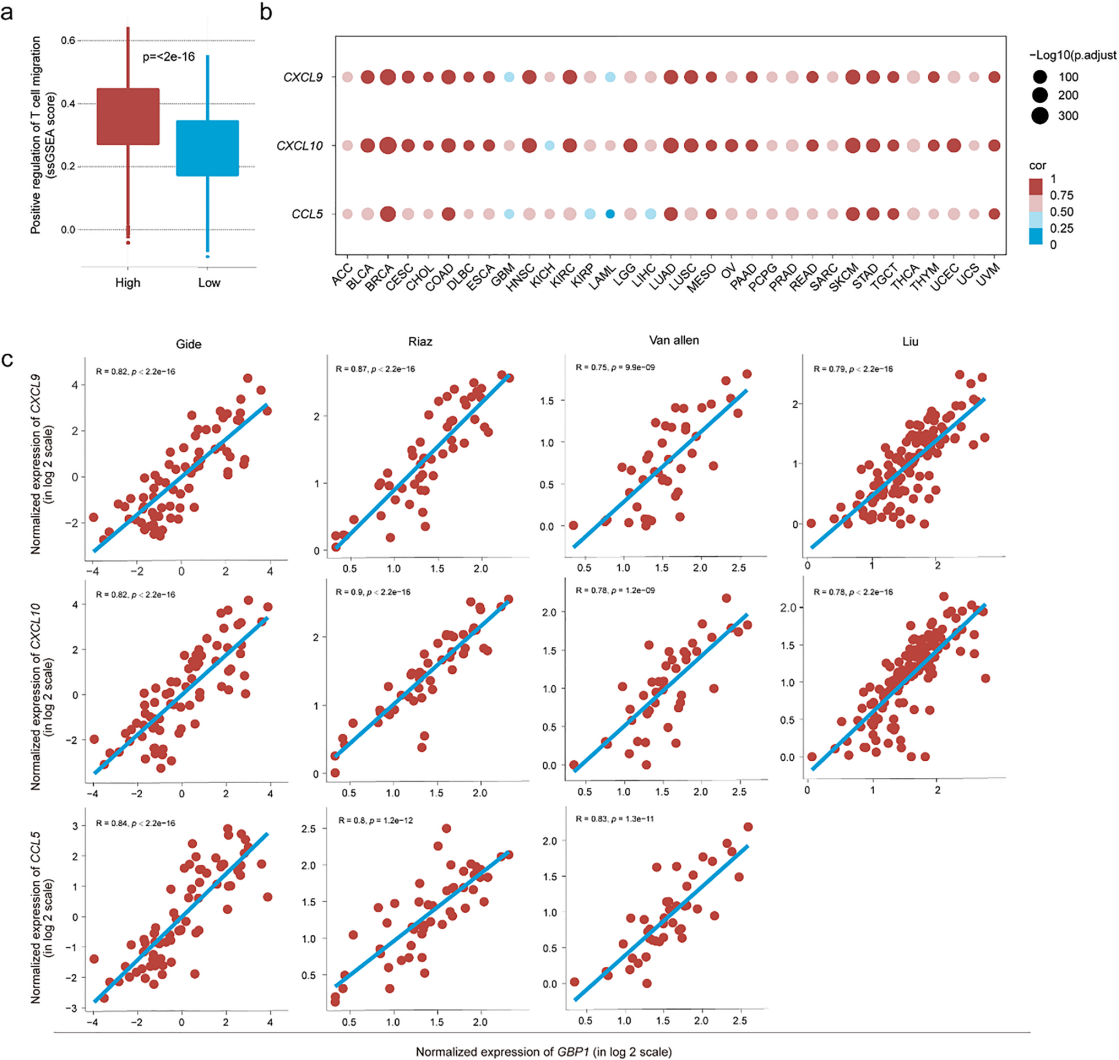
Correlation between *GBP1* expression and key chemokines that direct CD8^+^ T-cell migration. **a** T-cell migration score between patients with high and low *GBP1* expression. **b** and **c** Correlation of *GBP1* expression and that of *CCL5*, *CXCL9*, and *CXCL10* in The Cancer Genome Atlas (TCGA) pan-cancer analysis (**b**) and immunotherapy cohorts (**c**)

T-cell migration to the TME is dependent on multiple chemokines, including CCL5, CXCL9, and CXCL10 [40, 41]. To confirm the relationship between GBP1 and T-cell migration, we compared T-cell migration scores based on *GBP1* expression. Patients with high *GBP1* expression displayed significantly higher T-cell migration scores (Fig. 5a). We then focused on the above three chemokines and found that *GBP1* was positively correlated with *CCL5*, *CXCL9*, and *CXCL10* across 33 cancer types in the TCGA (Fig. 5b). We analyzed immunotherapy cohorts and observed the same phenomenon (Fig. 5c). This suggests that the positive correlation between *GBP1* and the key chemokines was consistent across multiple cohorts, indicating that GBP1 may recruit CD8^+^ T cells by enhancing the production of CCL5, CXCL9, and CXCL10.

### GBP1 expression is related to CD8^+^ T-cell infiltration and response to immune checkpoint blockade in clinical cohorts

**Fig. 6:**
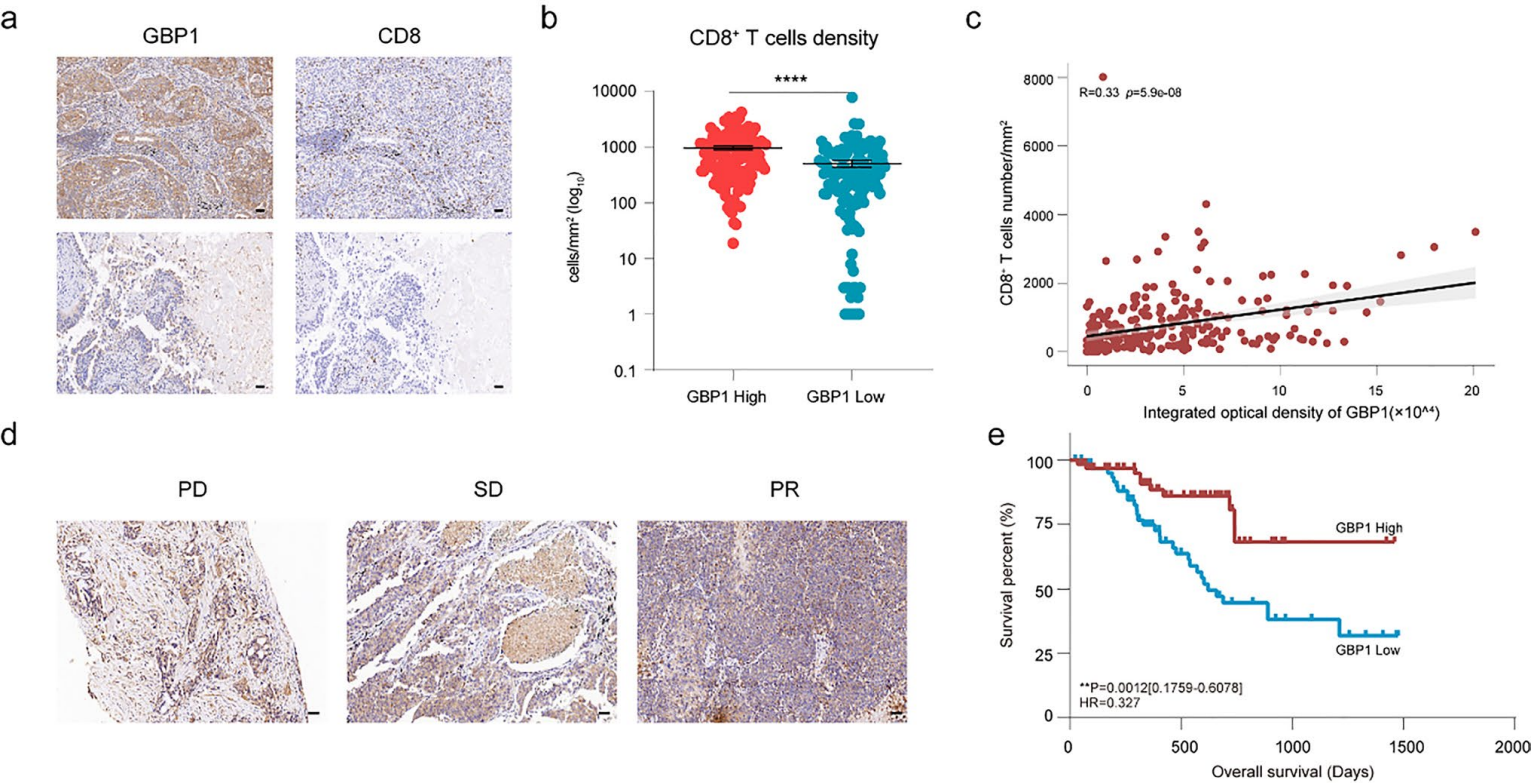
GBP1 expression was related to CD8^+^ T-cell infiltration and the response to immune checkpoint blockade in clinical cohorts. **a** Representative images of GBP1 expression and CD8^+^ T-cell infiltration in cohort 1. Scale bar: 50 μm. **b** Density of CD8^+^ T cells in the high and low GBP1 expression groups. **c** Correlation between GBP1 expression and CD8^+^ T-cell infiltration in cohort 1. **d** Representative immunohistochemistry (IHC) staining images of GBP1 from patients with different treatment responses in cohort 2. Scale bar: 50 μm. **e** Overall survival analysis of patients in cohort 2 based on GBP1 expression

The abovementioned results suggest a positive role for GBP1 in anti-tumor immunity and immunotherapy in multiple tumor types. To validate these findings in clinical samples, we collected two cohorts of lung cancer tissues to construct tissue microarrays. GBP1 expression and CD8^+^ T-cell infiltration were detected via immunohistochemistry (IHC) analysis. In cohort 1, patients were divided into high and low GBP1 expression groups. CD8^+^ T-cell infiltration was significantly higher in the high GBP1 expression group (Fig. 6a and b). Correlation analysis revealed that GBP1 expression was positively correlated with CD8^+^ T-cell infiltration (Fig. 6c). This is in accordance with the previous bioinformatics findings. This result supports previous findings that GBP1 is likely an indicator of CD8^+^ T-cell infiltration.

To validate the association between GBP1 expression and treatment response and survival after immunotherapy, we further analyzed the relationship between GBP1 expression and clinical response in cohort 2. The results suggested that GBP1 expression staining in biopsies from patients with PR or SD was higher than in those from patients with PD (Fig. 6d). The patients were stratified into high and low GBP1 expression groups based on the median expression of GBP1. Overall survival of the high GBP1 expression group was also significantly longer than that of the low-expression group (HR = 0.327 [0.1759–0.6078], *p* = 0.0012) (Fig. 6e). These results are in accordance with those of the previous analyses of public data and indicate that GBP1 could serve as a potential prognostic marker for immunotherapy.

## Discussion

It is becoming increasingly clear that immune responses to ICB are influenced by multiple factors, including tumor mutations and immune response biomarkers. PD-L1, TMB alone, or these in combination have been tested as predictive biomarkers in clinical trials and showed promising results [42–44]. Although these biomarkers have been correlated with responses to ICB, problems still exist. Standardization of PD-L1 staining and the optimal cutoff of TMB led to incomplete enrichment of responses. In addition, the multitude of networks and cellular phenotypes in the TME have not been considered. Ayers et al. developed T-cell-inflamed gene expression profiles (GEPs) related to antigen presentation, chemokine expression, cytotoxic activity, and adaptive immune resistance [23]. This immune signature was validated in KEYNOTE-028 and could predict the response to pembrolizumab in multiple tumor types [45]. However, optimization in terms of GEP calculations in individual patients and the economic cost also hinder its use in a clinical setting. Therefore, there is an increased demand for using currently available methods to develop a single gene with immediate translational potential.

In this study, we initially investigated three immune-related pathways (IFN-α/β/IFN-γ/IL-15 pathways) that are involved in the functional development of CD8^+^ T cells [11]. Type I IFN is released by almost all cells in the body, and cancer cells serve as one of its sources in tumors [46, 47]. It plays a critical role in anti-tumor immunity by supporting CD8^+^ T-cell activation [9, 48]. The previous studies identified type I IFN as a predictive biomarker in patients with breast carcinoma that was treated with anthracycline-based chemotherapy [49]. Recombinant type I IFN has also been approved as an anticancer treatment [50, 51]. However, the role of type I IFN in predicting immunotherapy response and survival has not yet been reported. IFN-γ is a primary effector cytokine of cytotoxic CD8^+^ T cells, which has been reported to highly correlate with clinical response to therapy in melanoma [52] and breast cancer [53]. IL-15 is a cytokine primarily released by dendritic cells, monocytes, and epithelial cells. It functions as a critical factor for homeostatic proliferation and survival of memory CD8^+^ T cells [12]. Combination therapy of IL-15 with anticancer monoclonal antibodies has entered clinical trials [54]. The current findings suggest that IFN-α/β/IFN-γ/IL-15 pathways may serve as predictive markers in immunotherapy. Thus, we explored their predictive effect in the immunotherapy cohorts. As expected, patients with higher expression levels exhibited better clinical responses and survival. These results demonstrated that the IFN-α/β/IFN-γ/IL-15 pathways likely confer sensitivity to ICB and could serve as predictive markers.

However, it is inconvenient and costly to detect all the molecules involved in these pathways in the clinic. Therefore, we investigated whether a single gene could represent these pathways. Based on the TCGA project data, we identified *GBP1* as a gene positively correlated with the IFN-α/β/IFN-γ/IL-15 pathways, which indicated that *GBP1* could specifically represent these pathways. As IFN-α/β/IFN-γ/IL-15 pathways are involved in the activation and memory of CD8^+^ T cells [11], we analyzed the relationship of *GBP1* with CD8^+^ T cells in the TME. *GBP1* was significantly associated with CD8^+^ T-cell infiltration and the hot TME. To confirm the above results, we stained CD8^+^ T cells in tissue microarrays of lung cancer. A positive correlation between GBP1 expression and CD8^+^ T-cell infiltration was observed. These results indicate that GBP1 could reflect the infiltration of CD8^+^ T cells in patients.

Survival analysis revealed that higher expression of *GBP1* improved survival rates. The number of responders was much higher in the high *GBP1* expression group. These results were in accordance with analysis of the IFN-α/β/IFN-γ/IL-15 pathways. We found a comparable predictive effect of *GBP1* with that of previously reported signatures and biomarkers. In addition, GBP1 is a single biomarker and could more easily be detected with IHC than with TMB, T-cell clonality, etc. To test the predictive effect of GBP1, we conducted GBP1 staining in tissue microarrays of patients with lung cancer who had been treated with immunotherapy. Based on the expression level of GBP1, we found that the overall survival was also longer in the high GBP1 expression group. These results support the potential of using GBP1 as a biomarker.

*GBP1* is a critical IFN-stimulated gene that can be activated by type I or II IFNs and plays an important role in innate immunity [34]. As GBP1 can be induced by IFN signaling, patients with high GBP1 expression may reflect active IFN signaling, which may represent the active anti-tumor immunity. The previous studies found that PD-L1 expression is frequently driven by IFN-γ exposure, which type I IFNs can also induce [55]. Intratumoral PD-L1 expression can be considered an indirect measure of local T-cell activity [56]. However, this reflects only the transient status. GBP1 has been reported to moderate the reaction to IFN signaling and inhibit cellular sensitivity to apoptosis in response to inflammatory stimuli [57]. This may support long-term T-cell function, which is in accordance with the function of the IL-15 pathway. The expression of GBP1 may not only reflect the activation of CD8^+^ T cells, but may also reveal the sustained effect of CD8^+^ T cells. Thus, GBP1 could more comprehensively reflect the functional role of CD8^+^ T cells than PD-L1 can. However, the role of GBP1 in anti-tumor immunity has not yet been reported. In addition, some studies have shown that tumor cells expressing GBP1 can promote tumor growth [58]. Thus, the observed predictive effect of GBP1 raised the question of which cells expressed GBP1, and how they contribute to anti-tumor immunity. Single-cell datasets were used to analyze the cell distribution of *GBP1*. It was found that *GBP1* was mainly expressed in macrophages but not tumor cells. Macrophages are a heterogeneous subtype that includes anti-tumor M1 and pro-tumor M2 macrophages [59]. The predominant expression of GBP1 in macrophages indicates that it supports their anti-tumor role. GSEA revealed that T-cell migration and activation signaling were enriched. The previous studies have shown that macrophages produce CXCL9 and CXCL10, which are key chemokines that direct CD8^+^ T cells [60]. A recent study also demonstrated that macrophage-derived CXCL9 and CXCL10 are pivotal for ICB [39]. Our study found a strong correlation between GBP1 and chemokines that direct T cells. These results suggest that GBP1 in tumors may promote chemokine production, thus enhancing CD8^+^ T-cell infiltration.

In summary, this study aimed to identify a gene that reflects the entire course of CD8^+^ T-cell development during anti-tumor immunity. IFN-α/β/IFN-γ/IL-15 pathways were chosen for initial analysis, and we found that *GBP1* could represent them. The predictive effects of GBP1 were tested and validated in discovery and validation cohorts. This study comprehensively analyzed the role and potential function of GBP1 in anti-tumor immunity and immunotherapy. Further studies are needed to investigate how GBP1 influences CD8^+^ T-cell recruitment and its potential role as a therapeutic target.

## Supplementary Information

Below is the link to the electronic supplementary material.

Supplementary file1 (DOCX 984 kb)

## Data Availability

All relevant data analyzed during this study were included in the methods section of this article.

## Abbreviations

AUC: Area under the receiver operating characteristic curve
DEGs: Differentially expressed genes
DAB: Diaminobenzidine
GBP1: Guanylate-binding protein 1
GEO: Gene Expression Omnibus
GEPs: Gene expression profiles
ICI: Immune checkpoint inhibitor
ICB: Immune checkpoint blockade
IHC: Immunohistochemistry
MDSCs: Myeloid-derived suppressor cells
NSCLC: Non-small cell lung cancer
ORR: Objective response rate
OS: Overall survival
TAMs: Tumor-associated macrophages
TCGA: The Cancer Genome Atlas
TMB: Tumor mutational burden
TME: Tumor microenvironment
UMAP: Uniform Manifold Approximation and Projection
