# Supplementary material for: IFN-α/β/IFN-γ/IL-15 pathways identify GBP1-expressing tumors with an immune-responsive phenotype
Source: Clin Exp Med. 2024 May 17;24(1):102. doi: 10.1007/s10238-024-01328-w (PMC11101573; doi:10.1007/s10238-024-01328-w)

**Title: IFN-α/β/IFN-γ/IL-15 pathways identify GBP1-expressing tumors with an immune-responsive phenotype**

**Journal: Clinical and Experimental Medicine**

Lei Wang^a*^, Yuxuan Wei^a*^, Zheng Jin^b,c*^, Fangfang Liu^a^, Xuchang Li^a^, Xiao Zhang^d^, Xiumei Bai^d^, Qingzhu Jia^e,f^, Bo Zhu^e,f^, Qian Chu^a^

^a^Department of Oncology, Tongji Hospital, Huazhong University of Science and Technology, Wuhan, Hubei 430030, P.R. China.

^b^Institute of Life Sciences, Chongqing Medical University, Chongqing, 400032, P.R. China.

^c^Research Institute, GloriousMed Clinical Laboratory (Shanghai) Co.,Ltd, Shanghai, 201318, P.R. China.

^d^Army 953 Hospital, Shigatse Branch of Xinqiao Hospital, Army Medical University, Shigatse, 857000, P.R. China.

^e^Department of Oncology, Xinqiao Hospital, Army Medical University, Chongqing 400037, P.R. China.

^f^ Chongqing Key Laboratory of Immunotherapy, Chongqing 400037, P. R. China.

*Lei Wang, Yuxuan Wei and Zheng Jin are contributed equally to this study.

**Correspondence:**

Qian Chu, E-mail: qianchu@tjh.tjmu.edu.cn.

**Supplement Figure S1** The overall survival was analyzed when IFN-α/β/IFN-γ/IL-15 pathways were all high expressed in patients

**Supplement Figure S2** The correlation between *GBP1* expression and IFN-α/β/IFN-γ/IL-15 pathways was analyzed in four GEO datasets: GSE81089, GSE103584, GSE112996, and GSE181820

**Supplement Figure S3** The genes that differed between *GBP1* high and low groups were analyzed and used for enrichment analysis

**Supplement Figure S4** The cell specific expression of *GBP1* in non-small cell lung cancer and melanoma single cell datasets

Supplement Figure S1


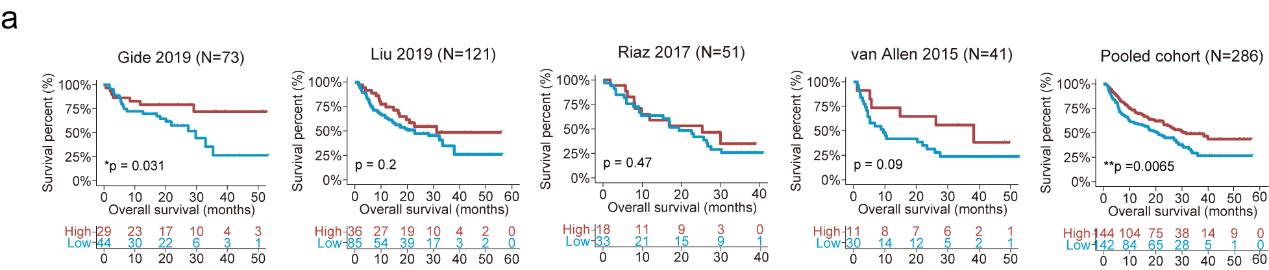


Supplement Figure S2


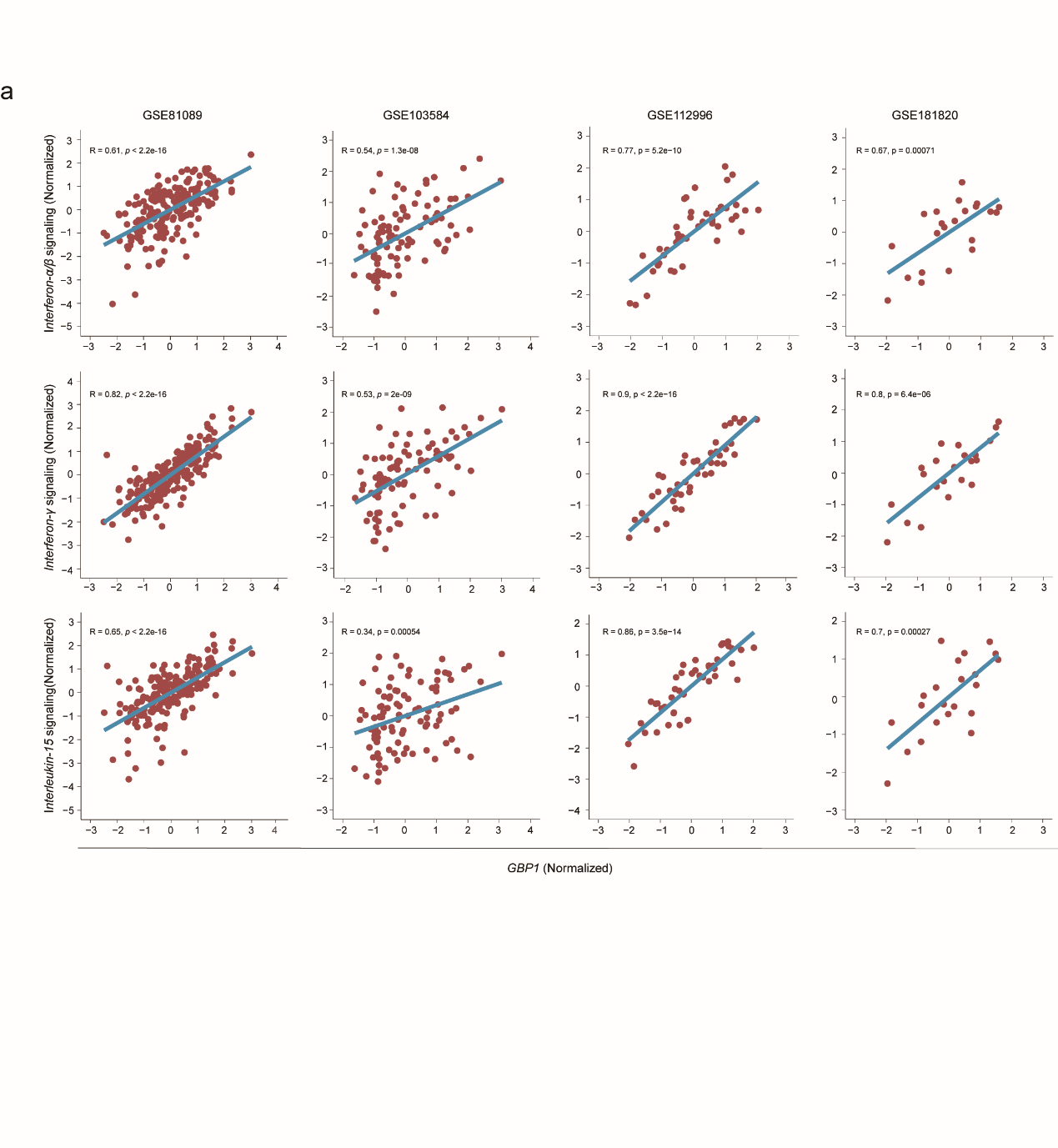


Supplement Figure S3


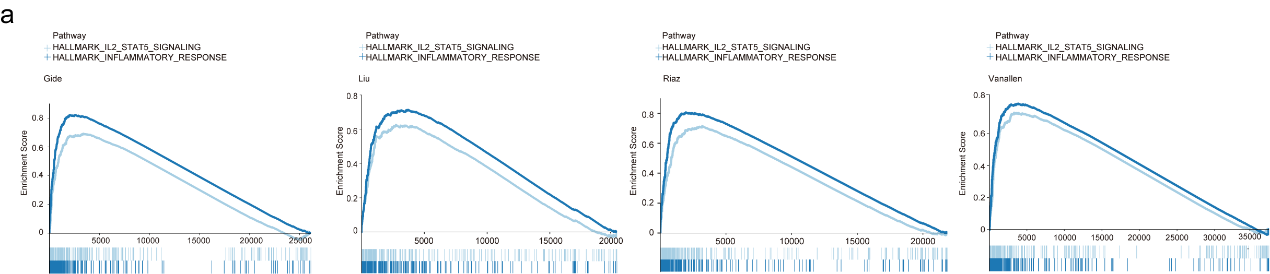


Supplement Figure S4


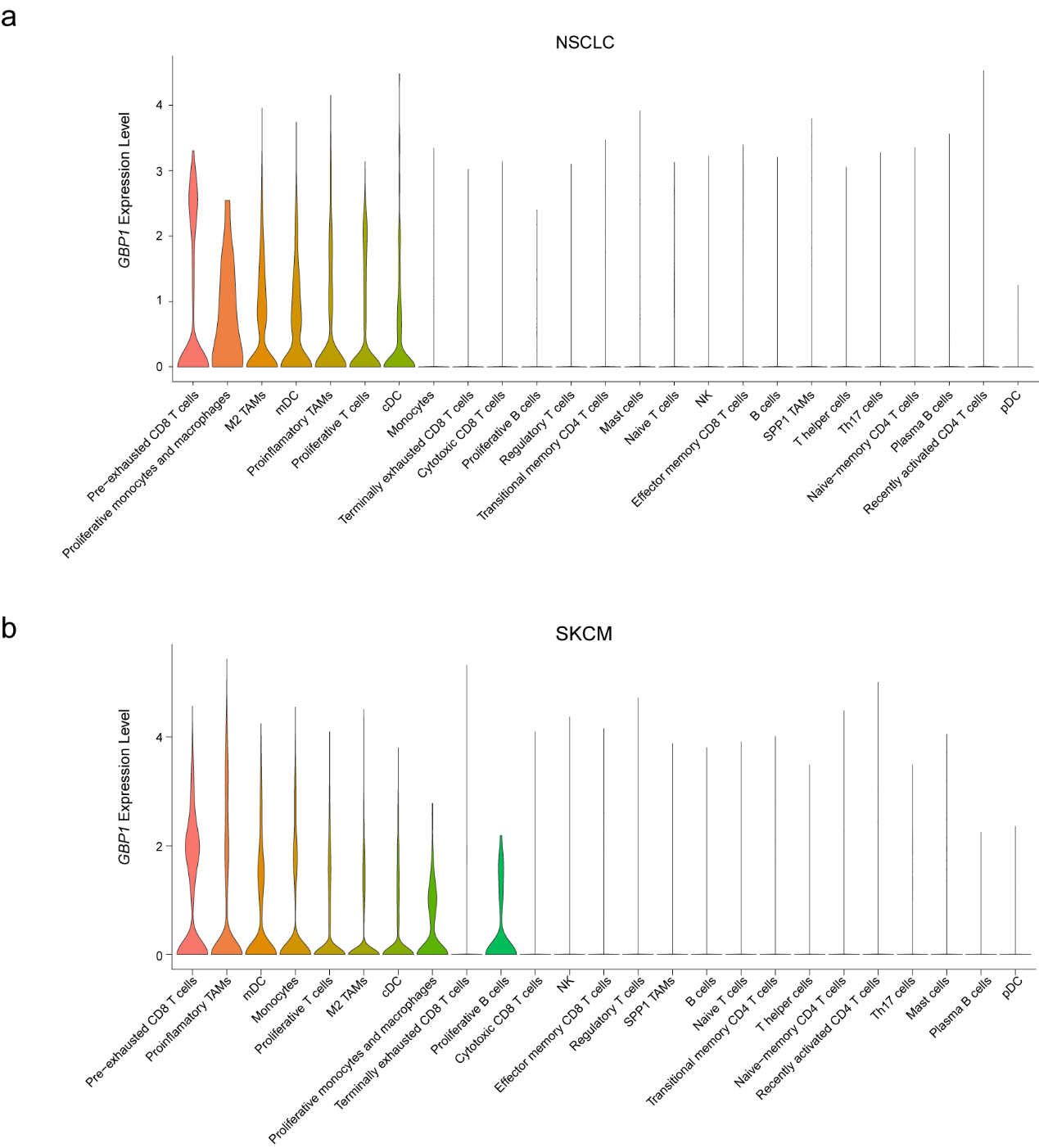

Supplement: Supplementary file 1 — Supplementary file1 (DOCX 984 kb) [file 10238_2024_1328_MOESM1_ESM.docx]
